# Experimental Seaborne Passive Radar

**DOI:** 10.3390/s21062171

**Published:** 2021-03-20

**Authors:** Gustaw Mazurek, Krzysztof Kulpa, Mateusz Malanowski, Aleksander Droszcz

**Affiliations:** Faculty of Electronics and Information Technology, Warsaw University of Technology, Nowowiejska 15/19 Street, 00-665 Warsaw, Poland; k.kulpa@ise.pw.edu.pl (K.K.); aleksander.droszcz.stud@pw.edu.pl (A.D.)

**Keywords:** passive bistatic radar, passive coherent location, maritime, seaborne

## Abstract

Passive bistatic radar does not emit energy by itself but relies on the energy emitted by illuminators of opportunity, such as radio or television transmitters. Ground-based passive radars are relatively well-developed, as numerous demonstrators and operational systems are being built. Passive radar on a moving platform, however, is a relatively new field. In this paper, an experimental seaborne passive radar system is presented. The radar uses digital radio (DAB) and digital television (DVB-T) for target detection. Results of clutter analysis are presented, as well as detections of real-life targets.

## 1. Introduction

Passive coherent location (PCL) radar, also known as passive bistatic radar, relies on the signals emitted by illuminators of opportunity, such as radio or television transmitters [1]. The radar is equipped with at least two receiving channels. One channel is connected to an antenna that is pointing towards the transmitter. In this way, the reference signal is received. The second channel is connected to an antenna that is used to receive the echo signal. By correlating the reference and echo signals, one can estimate the time delay and frequency shift. The system can localize the target in the Cartesian coordinates by combining the delay and frequency shift measurements from multiple transmitter–receiver pairs. As a result, passive radar can be effectively used for the tracking of moving targets.

Most passive radars are stationary ground-based systems. The technology is relatively mature and well-understood. One of the challenges in passive radar technology is installing passive radar on moving platforms. This includes airplanes [2,3,4,5,6], ground vehicles [7], and ships [8,9].

In this paper, we focus on seaborne passive radar, where the system was installed on a ship. First, the passive radar system is presented. Next, the processing algorithms are introduced. Finally, the real-life clutter analysis and the results of the experiments on the detection of airborne targets using digital radio (DAB) and digital television (DVB-T) are shown. The measurement campaign in which we collected the data was the NATO APART-GAS 2019 (active passive radar trials ground-based, airborne, seaborne) trials, which occurred in Poland on the Baltic Sea in September 2019 [10,11,12,13]. The trial involved numerous cooperative targets, such as aircrafts and ships, and various radar sensors, including passive and active systems either stationary or installed onboard different moving platforms.

The paper’s novelty is the presentation of real-life clutter characteristics during different sea states and the detection of cooperative and noncooperative airborne targets by seaborne passive radar, which confirms its usefulness.

## 2. Materials and Methods

### 2.1. System Setup and Signal Acquisition

**Figure 1:**
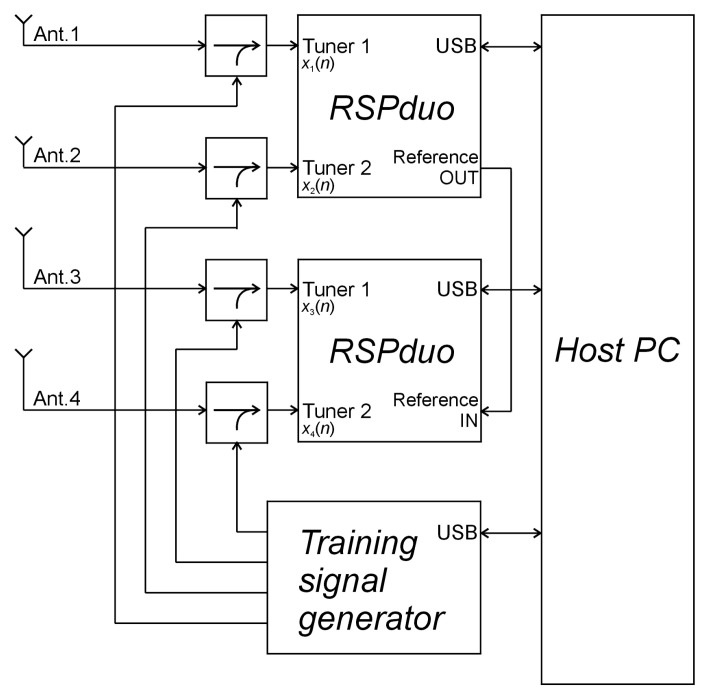
Four-channel receiver used in the experiments.

A block diagram of the considered PCL receiver is depicted in Figure 1. The hardware setup consists of two dual-channel software-defined radio (SDR) receivers (RSPduo [14]) connected to a host PC, a receiving antenna array, and a training signal generator. All tuners in the receivers are tuned to the same frequency of a DAB or DVB-T broadcast station selected for the illumination of a radar scene. The RSPduo devices are connected to the host PC via distinct USB ports used for data transfer and power supply. The training signal generator is controlled and powered from another USB port. It is responsible for the periodical generation of pilot signals that are later employed in the synchronization of the channels [15]. The pilot signals are combined with RF signals from the receiving antennas using a set of directional couplers. After filtering, downconversion, and digitalization in the RSPduo devices, the received complex baseband signals are finally stored in the SSD drive of the host PC for further offline processing.

**Figure 2:**
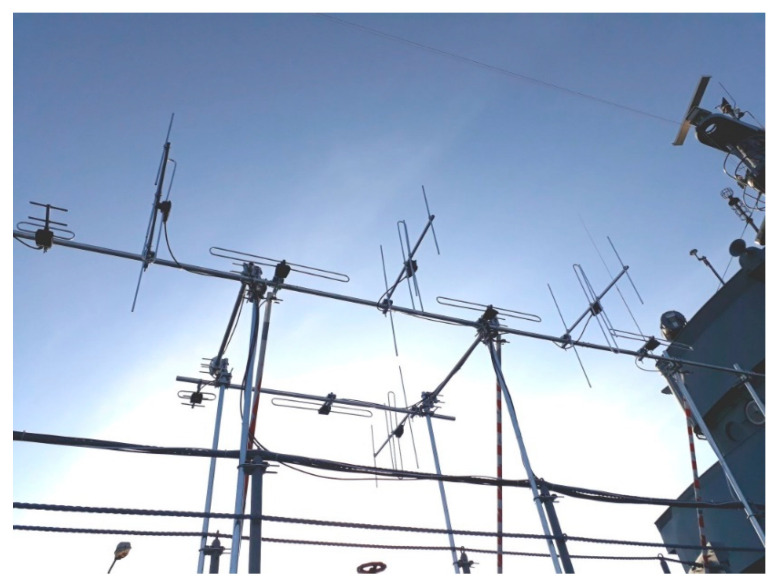
Receiving antenna array installed on the side of the ship.

**Figure 3:**
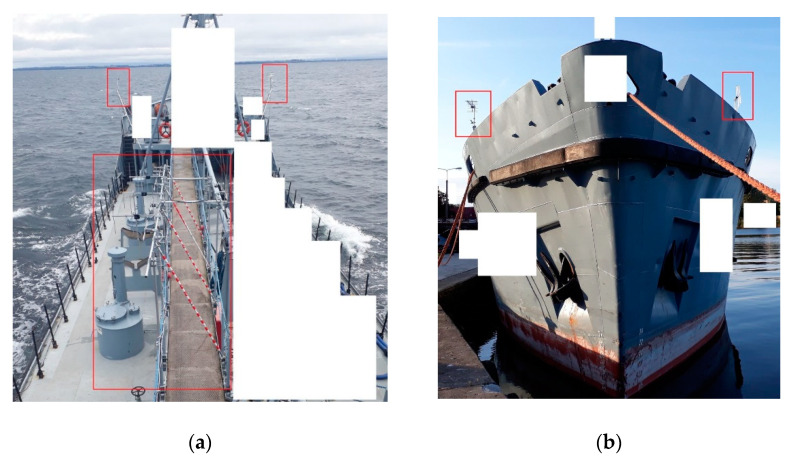
Top view of the antenna array (**a**) and additional reference antennas on front of ship (**b**) (some parts of the picture are masked for security and privacy reasons).

The receiving antenna array installed for PCL experiments on the port side of the ship is shown in Figure 2 and Figure 3 with the proviso that only part of the antennas were used in the experiments described in this paper. Four commercial-grade VHF antennas (Yagi–Uda, 800 × 100 × 750 mm, 4-element, 6 … 8 dBi gain, vertical polarization) were employed to acquire RF signals from the DAB frequency band; three of them in the surveillance path and one in the reference path—installed in the opposite direction. Another set of four antennas (dipole, 200 × 700 × 100 mm, 2 dBi gain, horizontal polarization) was installed for DVB-T reception. An additional vertically polarized VHF antenna was installed on the front of the ship as a reference, as shown in Figure 3. The surveillance antenna set and the reference antenna were selected individually for each signal acquisition, depending on the experiment’s scenario.

A DAB transmitter (218.64 MHz, 1.536 MHz bandwidth, 3.2 kW ERP, vertical polarization, 54°12′15″ N, 16°13′42″ E) and a DVB-T transmitter (191.5 MHz, 7.6 MHz bandwidth, 15 kW ERP, horizontal polarization, 54°00′15″ N, 16°44′27″ E) were selected as the sources of illumination, both located near Koszalin, Poland (ca. 15 km from the coast). Those transmitters were chosen because of their vicinity to the measurement area. An interesting fact is that DVB-T transmitters usually operate in the UHF band (approximately 400–800 MHz). In Poland, however, some DVB-T transmitters also operate in the VHF band (around 200 MHz), which provided an interesting and unusual opportunity for measurement. Between consecutive signal acquisition sessions, the PCL receiver was alternately tuned to either the DAB or DVB-T channel. In each case, a set of antennas with polarization consistent with the illuminating transmitter was employed.

**Figure 4:**
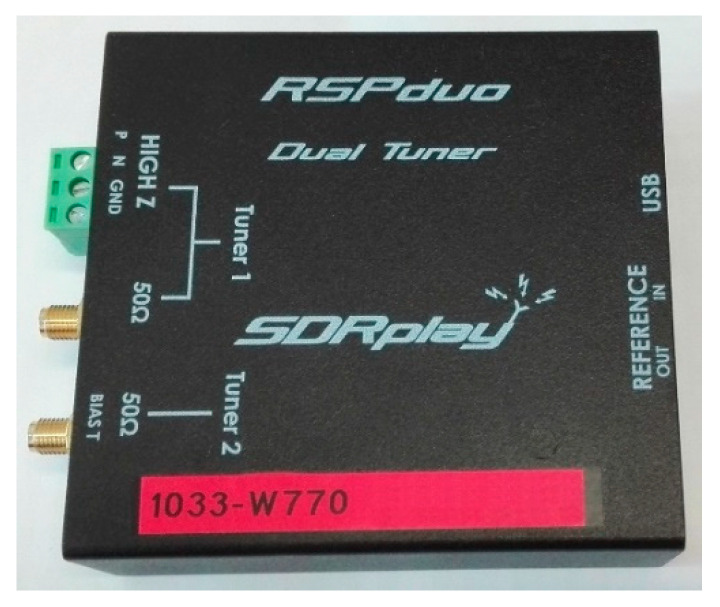
RSPduo: dual-tuner SDR (software-defined radio) receiver.

The RF signals were downconverted and digitized in two RSPduo devices (shown in Figure 4), which are compact dual-tuner SDR receivers from SDRplay [14], intended for general purpose SDR applications in the frequency range from 1 kHz up to 2 GHz [16]. The sampling rate was set to *F*_s_ = 2 MHz, which allows it to receive the whole bandwidth of the DAB signal. In the case of the DVB-T signal, only a part of the bandwidth is acquired in this way. The remaining out-of-band components are filtered out through the RSPduo tuner, thanks to internal oversampling and anti-aliasing filters.

Each RSPduo device was connected to the host PC via a High-Speed USB 2.0 interface (also used as the power supply) and contained two complete RF frontends. Each frontend included a bank of bandpass filters, variable-gain LNA (low-noise amplifiers), and optional notch filters. The ADC (analog-to-digital converter) had 14-bit performance (for a 2 MSPS sample rate), and the sensitivity was around 0.1 μV [16]. The receiver’s performance, combined with the completeness of the RF frontend, the small size of the device, and the low power consumption, determined the choice of the device for our experiments.

Two or more RSPduo devices can be synchronized by connecting their dedicated reference OUT/IN ports, so it is possible to obtain the same sampling rates and carrier frequencies in multiple RF tuners. However, providing repeatable, coherent multichannel signal acquisition requires additional hardware and software modules [15], such the training signal generator (see Figure 1).

**Figure 5:**
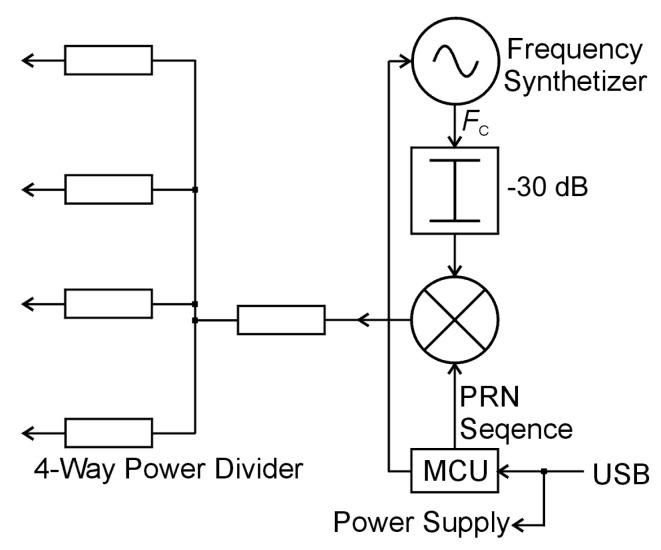
Internal structure of the training signal generator.

Due to the lack of a common trigger signal, the coherent acquisition from two or more RSPduo devices becomes a challenge [17]. Each of the devices starts signal reception with an unknown delay (because of sequential initialization in the software) and a random phase shift due to the uncontrolled initial conditions of PLLs (phase-locked loops) in the downconverters. To solve this problem, a method for estimating phase shifts and time delays introduced in the receivers has been developed and implemented [15] based on the matched filtering of signals with superimposed pilot sequences from the training signal generator, whose architecture is shown in Figure 5.

The pilot sequence consisted of three repetitions of a complete PRN (pseudo-random) sequence, generated in an MCU (microcontroller) that modulates the carrier signal obtained from the frequency synthesizer followed by an attenuator. The carrier frequency (Fc) was programmed in accordance with the frequency of the broadcast transmitter used as the illumination source, and it was generated only during the short periods of pilot signal emission to avoid interferences with the received signals. BPSK (bipolar phase-shift keying) modulation was performed in a dual-balanced mixer. The modulated signal was then passed to a simple power divider to obtain its four copies that are finally fed into the directional couplers (see Figure 1.) The pilot signal was injected periodically in every 5 s.

**Figure 6:**
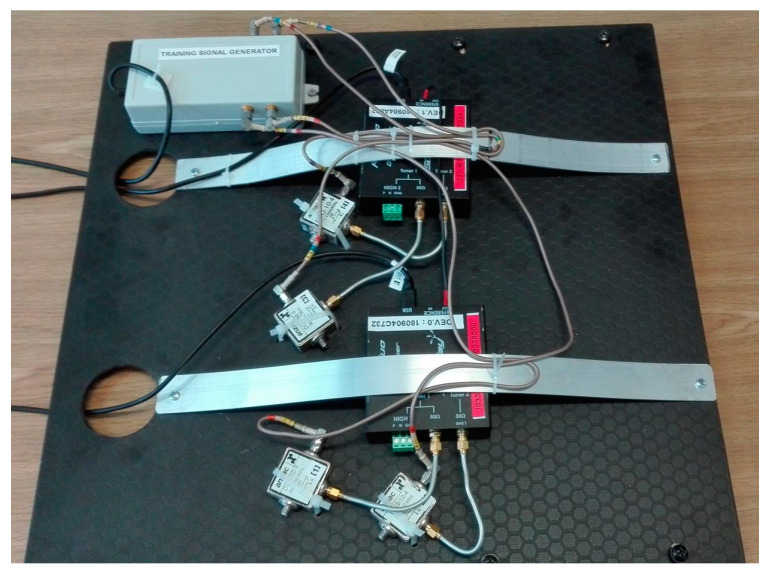
Four-channel receiver with the training signal generator and the directional couplers.

The acquired signals were stored on an SSD in the host PC, and were later processed offline in the MATLAB environment. During the preprocessing stage, the pilot signals were extracted with matched filters and used to calibrate the phase offsets and time delays between the received signals. The details of the training signal properties, generation, and processing have been disclosed in [15]. The experimental hardware setup that was used during the experiments (without a host machine, which was a typical notebook) is presented in Figure 6.

### 2.2. Signal Processing

**Figure 7:**
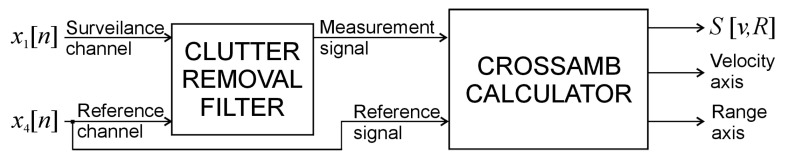
Simplified block diagram of the PCL (passive coherent location) processing algorithm.

The surveillance antennas were connected to channels 1, 2, 3 of the receiver and the reference antenna was connected to channel 4. After compensating for the phase shifts and time delays, the signals from the reference channel *x*_4_[*n*] and the selected surveillance channel *x*_1_[*n*] were processed using the PCL algorithm shown in Figure 7.

The remaining channels *x*_2_[*n*], *x*_3_[*n*] were intended to be processed by a more advanced version of the PCL algorithm supported by adaptive beamforming. The signal processing pipeline was composed of a clutter-removal filter (described with details in [1,18,19,20,21,22]) and a cross-ambiguity function calculator [1,23]. The default integration time processing algorithm was 262 ms. The final results of the processing are a series of 2-D plots *S*[*v*,*R*] showing signals reflected from the objects in the bistatic velocity/range (*v*/*R*) plane.

### 2.3. Compensation of Phase Fluctuations Caused by Ship’s Motion

**Figure 8:**
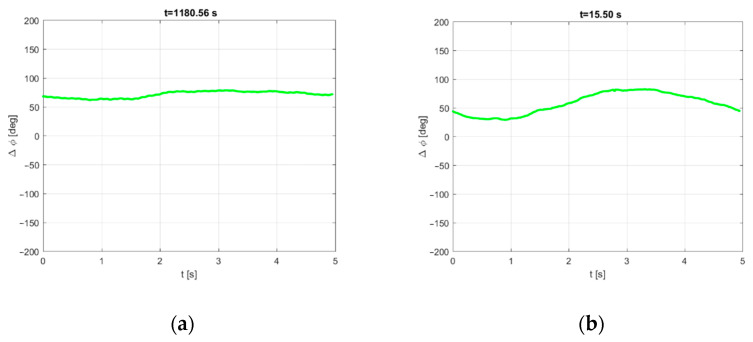
Surveillance/reference signals’ phase difference during calm seas (**a**) and the storm (**b**).

We must note that, during the storm, the ship swayed intensely in the rough waves and changed, to a small extent, its orientation relative to the illuminating transmitter. As the reference antenna was located at the front and the surveillance antennas were placed on the port side of the ship (see Figure 3), the distance between these antennas was several meters. As a result, both the phase and amplitude of the direct signal in the reference and surveillance antennas depended on the vessel’s pitch and roll, and the phase offset between the reference signal and its crosstalk in the measurement signal was changing during the integration time. On the other hand, the clutter removal filter requires a constant phase relationship between these signals during a single block’s coherent processing, such as in stationary PCL installation. Figure 8 shows the example plots of the discussed phase offset, estimated with 2 ms time steps during one integration period—in the cases of calm seas and the storm.

**Figure 9:**
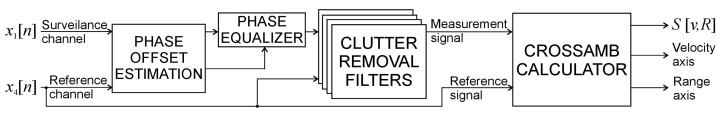
PCL processing algorithm modified for long integration periods.

The phase difference between the reference signal and its crosstalk fluctuated in both cases due to the ship’s movement. However, during the storm, these fluctuations were found to be much more intense. Our experiments with PCL signal processing have shown that these fluctuations severely degrade the clutter removal filter’s efficiency in the case of the long integration period. In such a case, because of the loss of coherency, it is almost impossible to filter out the direct path interference component and obtain clear PCL images. To address this problem, a more advanced signal processing algorithm has been developed with the structure presented in Figure 9.

Firstly, the phase offset between the reference signal and its crosstalk is estimated by computing a cross-correlation function on subsequent parts of the integration period (block size was 4000 samples, which corresponds to 2 ms). According to the obtained estimates, the phase offset of the measurement signal is then corrected in a phase equalizer, with spline interpolation on the entire integration period. After that, both signals are divided into 200 overlapping blocks of equal length. To improve immunity against time-variant signal properties, each of these blocks is processed in a separate instance of the clutter removal filter (described previously). Finally, the filters’ output signals are merged into the entire integration period and processed in a cross-ambiguity function calculator. This approach allows for practically removing the direct path interference component in severe time-varying phase offsets resulting from the rapid movement of the PCL platform. We employed such a modified PCL processing algorithm to obtain clutter images shown in the following section.

## 3. Results of Clutter Structure Analysis

**Figure 10:**
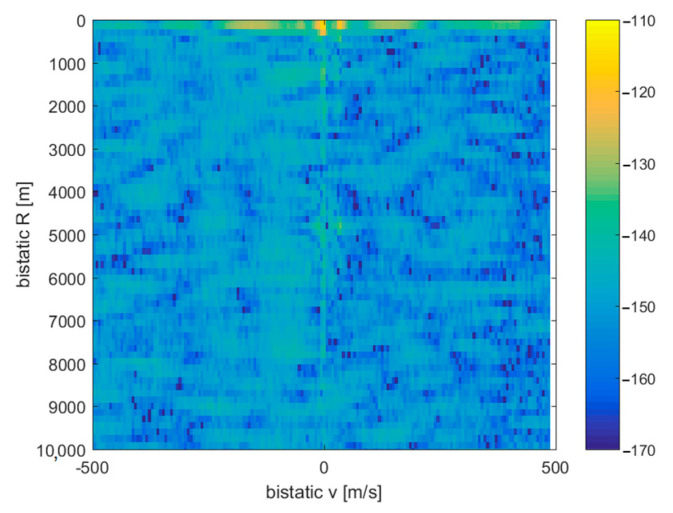
Sudden flash effect caused by the interference from an injected training signal.

Due to the training signals’ presence in the acquired signal, bright flashes occur in the whole *S*[*v*,*R*] plane, repeatable with *T*_r_ period, as shown in Figure 10. Since the training signal structure and its time of emission were known in advance, this interference may be removed using an adaptive filtering algorithm, for which development has been left for future work. Besides these short periods of interference, after filtering out the clutter, the cross-ambiguity plots can reveal the reflections from moving objects in the operating range of the system, as demonstrated in Section 4.

### Clutter Structure Variations

**Figure 11:**
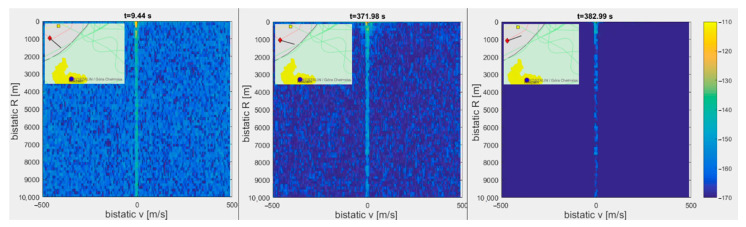
Change of clutter intensity caused by turning of platform with antenna array.

In moving PCL platforms, the residual clutter structure and intensity change as the platform is moving and changing its orientation [18,19,20,21]. In Figure 11, we can see that the ship is turning over a port, which changes the direction of the antenna array with respect to the illuminating transmitter. This change entailed a drop in the clutter intensity by 15 dB (the same color scale is used on three plots). In all cases, only the direct leakage signal was removed by an adaptive lattice clutter canceler [22] with the length of filtration truncated to the first two range cells.

**Figure 12:**
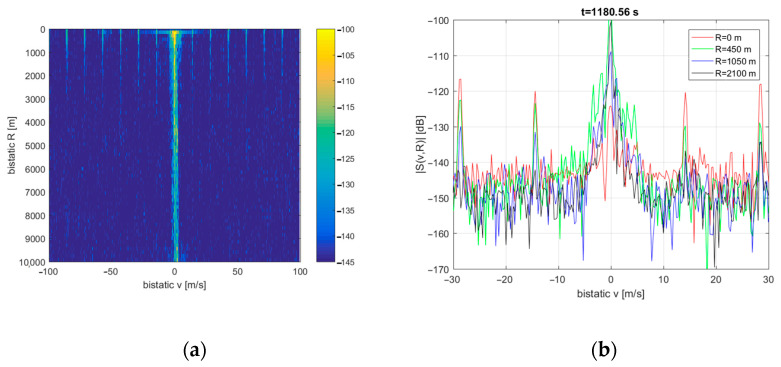
Clutter image during calm seas (**a**) and its cross-sections for selected bistatic ranges (**b**).

The residual clutter structure in marine PCL installation also depends on the sea state as determined by weather conditions. The clutter image observed during calm seas is depicted in Figure 12, with cross-sections for different bistatic ranges (*R*). For this experiment, the DAB transmitter (218.64 MHz) was selected for illumination, and the integration time was extended to 5 s to improve the resolution in Doppler frequency and bistatic velocity. The short parts (52 ms) of the signal with interference from the training sequence were excluded from each integrated block’s processing to obtain clear images.

With the increased integration time, we can see artifacts repeating in the bistatic velocity axis with a ca. 15 m/s period. These artifacts result from the periodical structure of the DAB signal observed in the time domain (i.e., the transmission frame duration of 96 ms in mode I [24] with the NULL symbol at the beginning of each frame). A method of mitigating this effect may be developed in further work to improve the image’s quality. Moreover, the clutter becomes Doppler-spread (up to 4 … 5 m/s) around *v* = 0 line due to the PCL platform’s motion.

**Figure 13:**
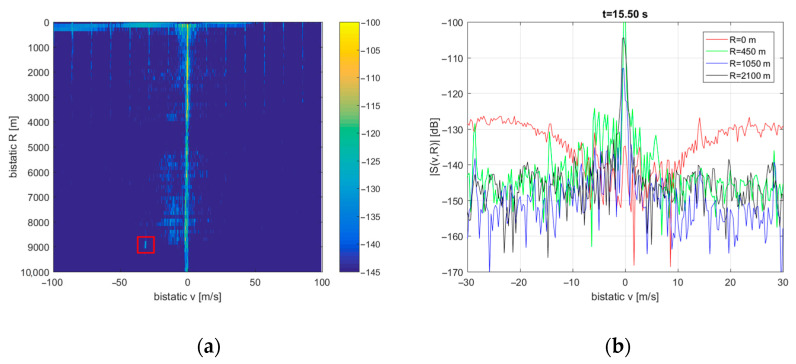
Clutter image during the storm (**a**) and its cross-sections for selected bistatic ranges (**b**).

A more distorted image of the residual clutter was obtained when PCL signal acquisition was performed during a storm (see Figure 13). The Doppler spread slightly increased for the nearest bistatic distances (e.g., *R* = 450 m). Additional regions of increased noise level (e.g., at *R* = 2 km, 4 km, 6 … 9 km) may result from signal reflections from heavy rainfall and rough waves at sea. In these regions, the Doppler spread can reach up to 10 … 20 m/s. The detection of departing CASA aircraft is also clearly visible around *R* = 9 km (the red rectangle). The echo is blurry in several range cells because of the long integration period in relation to the target’s speed. This aircraft detection proves that the modified version of the algorithm shown in Figure 9 can still detect moving targets.

## 4. Results of Moving Target Detection

A group of objects took part as cooperative targets in the APART-GAS campaign, such as civil and military aircraft, boats, and navy ships. In the following experiments, the integration time was set to 262 ms to facilitate the detection of fast-moving targets, unless noted otherwise. Target positions shown in maps have been derived from GPS logs and a separate ADS-B radio receiver.

### 4.1. Scenario 1: CASA C-295 and Fishing Boat (DVB-T Illumination)

**Figure 14:**
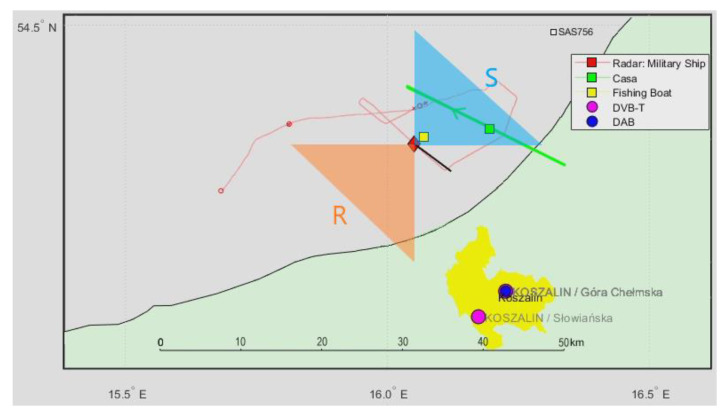
Situation map with a cooperative CASA C-295 aircraft and a nearby fishing boat.

In Figure 14, we can see a situation map with the military ship (carrying the PCL receiver) marked with a red square, a cooperative CASA C-295 aircraft [25] passing around 10 km from the ship (green mark), a cooperative fishing boat in the close surroundings (yellow mark), and an accidentally passing cruise plane SAS756 (white square). The boresight directions of the surveillance (S) and reference (R) antennas have been illustrated with triangles of different colors.

**Figure 15:**
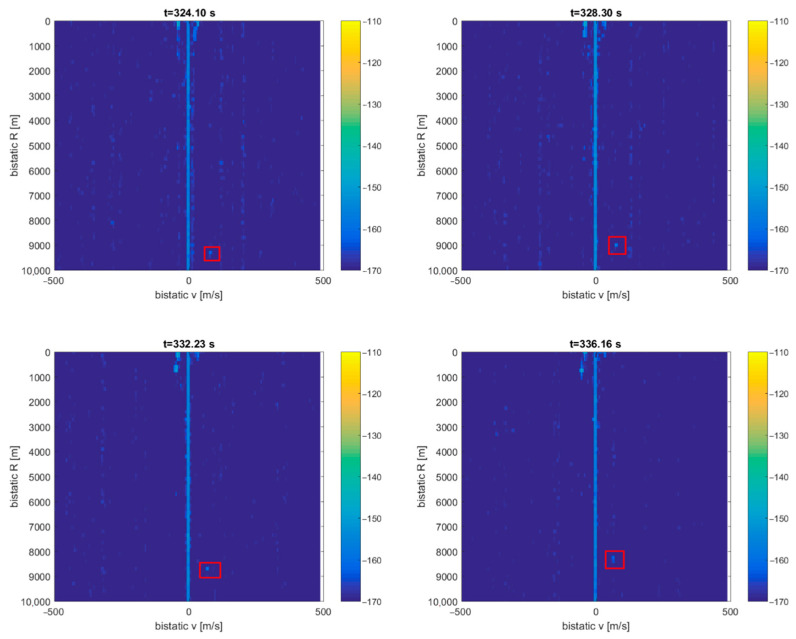
Detection results of CASA C-295 (subsequent time stamps), scene from Figure 14.

A signal from the DVB-T (191.5 MHz) transmitter was selected as a source of illumination with receiving antennas with horizontal polarization and the reference antenna from the side of the ship. After filtering out the clutter, the echoes from the CASA aircraft can be seen as a dot moving consequently in the bistatic *v*/*R* plane (around *R* = 9 km), marked with red rectangles in Figure 15 (the consecutive *v*/*R* maps are separated by 4 s). The fishing boat’s echoes from the close surrounding cannot be separated from the clutter image due to the negligible Doppler frequency. The echoes from the cruise plane SAS756 that was moving more than 20 km northeast from the ship are not visible on the plots.

### 4.2. Scenario 2: CASA C-295 and Fishing Boat (DAB Illumination)

**Figure 16:**
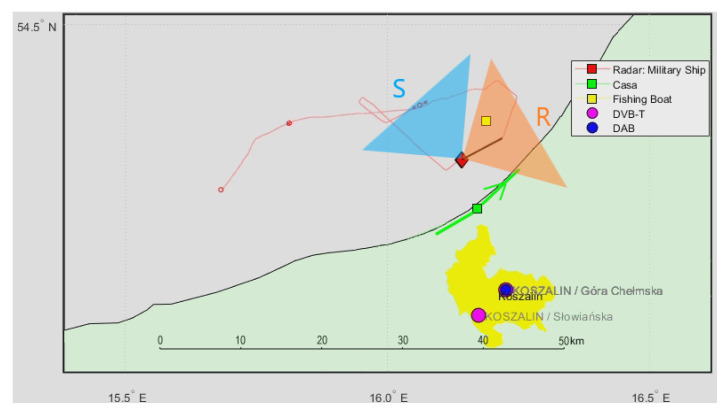
Situation map with a cooperative CASA C-295 aircraft and a distant fishing boat.

In Figure 16, we can see another situation map with the cooperative CASA C-295 aircraft passing on the starboard side of the ship, parallel to the coastline, between the illuminating transmitter and the PCL receiver, as well as the cooperative fishing boat around 8 km away from the ship. A signal from the DAB (218.64 MHz) transmitter has been selected for illumination with vertically polarized antennas and the reference antenna at the front of the ship.

**Figure 17:**
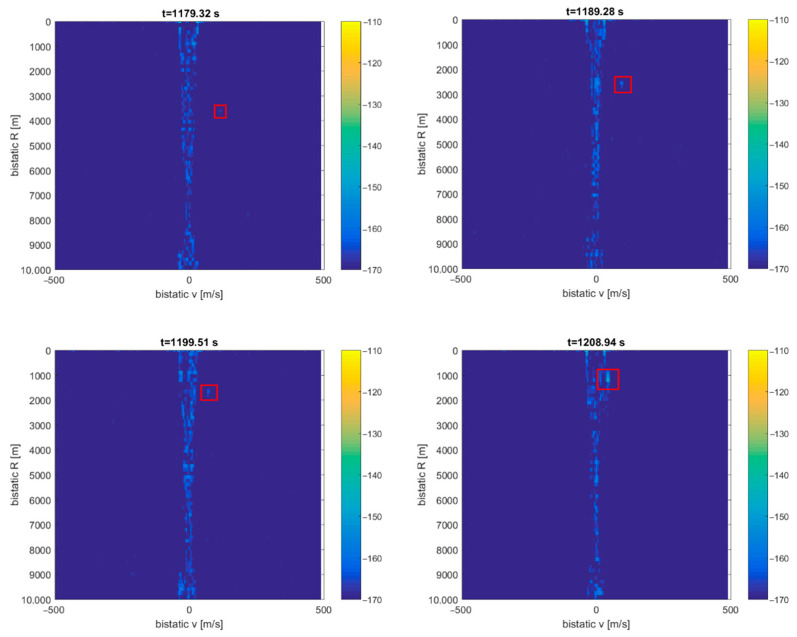
Detection results of the CASA C-295 aircraft (subsequent time stamps, scene in Figure 16).

The CASA aircraft’s detection results can be seen in Figure 17 as a dot moving in the bistatic *v*/*R* plane (from 3 to 1 km), marked with a red rectangle (the consecutive *v*/*R* maps are separated by 10 s). The intensity of this echo increases as the aircraft gets closer to the PCL receiver. The detection falls into the clutter area (*v* = 0) as the aircraft is flying above the transmitter–receiver baseline. The echoes from the fishing boat cannot be extracted from the plots since they are lost in the clutter.

### 4.3. Scenario 3: PC-12, Fishing Boat, CASA C-295, F-16 (DVB-T Illumination)

**Figure 18:**
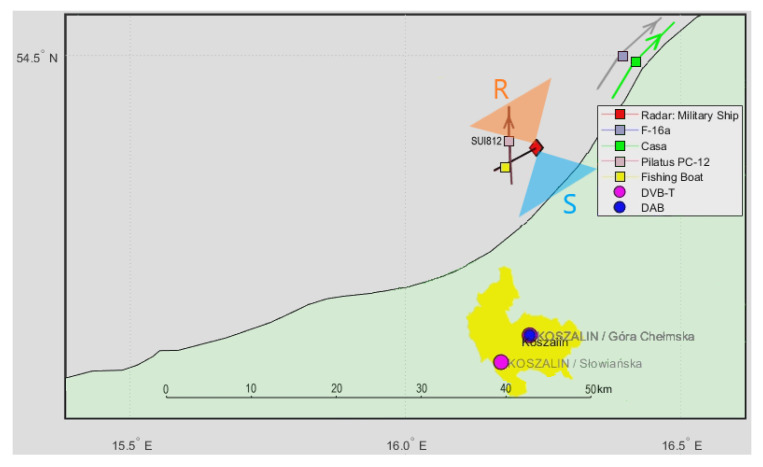
Situation map with the cooperative aircraft and fishing boat.

In Figure 18, we have shown a situation map with the cooperative PILATUS PC-12 (SUI812 [26]) aircraft passing in front of the ship, the fishing boat around 7 km from the front, and the CASA aircraft moving away at the back with an F-16 jet fighter. A signal from the DVB-T (191.5 MHz) transmitter was selected for illumination with horizontally polarized receiving antennas and the reference antenna from the side of the ship.

**Figure 19:**
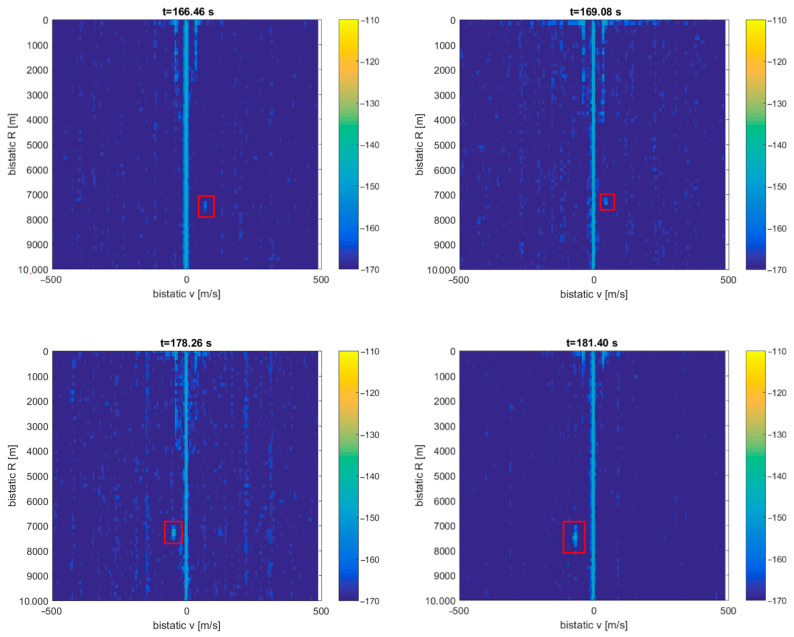
Detection results of the SUI812/PILATUS PC-12 aircraft (scene in Figure 18).

Detection results for this situation are depicted in Figure 19. The echoes from the PC-12 aircraft flying nearly the PCL receiver (ca. 5 km) are clearly seen in the bistatic *v*/*R* plane around *R* = 7.5 km, with the bistatic velocity turning from positive to negative. The detection was not possible during a period from 170 to 177 s when the bistatic velocity was close to zero and the echo was lost in the clutter. Detection of the fishing boat cannot be extracted from the *v*/*R* plots, and the distant F-16 jet fighter and CASA airplane are also not visible in this configuration.

### 4.4. Scenario 4: PC-12, Fishing Boat, F-16 (DVB-T Illumination)

**Figure 20:**
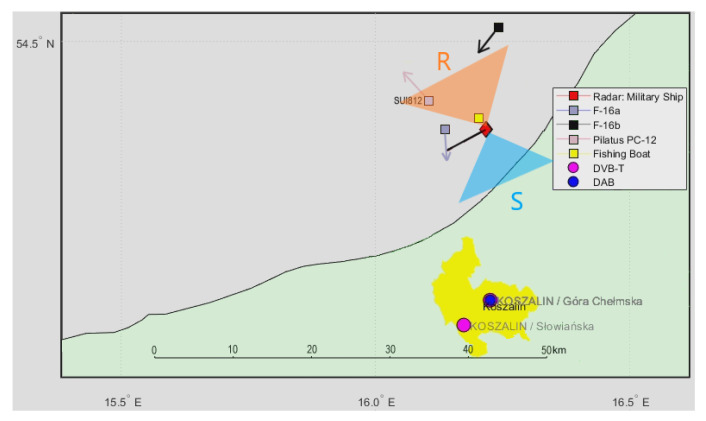
Situation map of the PILATUS PC-12 and two F-16 jet fighters.

A situation map with the PILATUS PC-12 aircraft (SUI812), two F-16 jet fighters flying near the ship, and the nearby fishing boat is shown in Figure 20 (PCL receiver setup was identical as previously.)

**Figure 21:**
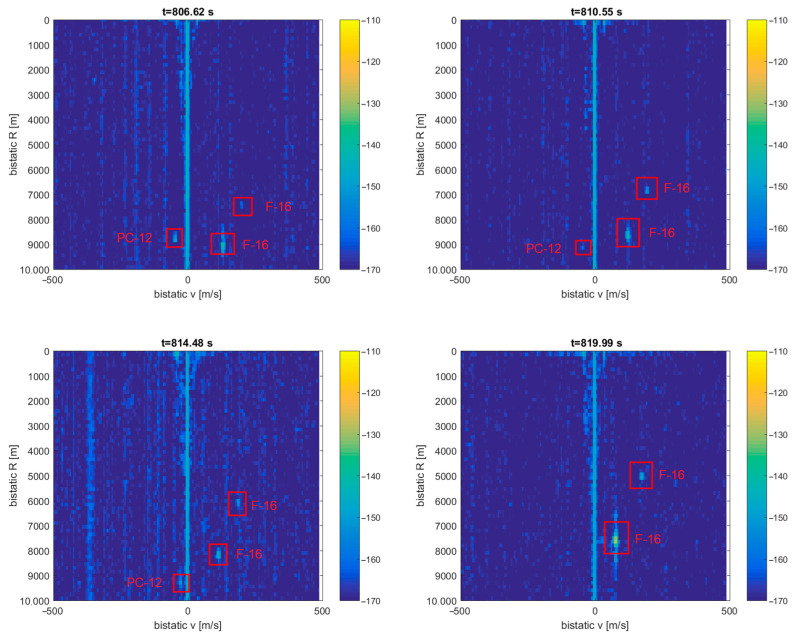
Detection results of the PILATUS PC-12 and two F-16 jet fighters (scene from Figure 20).

Detection results for this situation are depicted in Figure 21. The echoes from the PC-12 aircraft departing the PCL receiver are visible in the bistatic *v*/*R* plane around *R* = 9 km and moving to *v* = 0, *R* = 9.5 km. At the same time, we can see rapidly moving detection results from the two F-16 fighters. These echoes can move as fast as 200 m/s (in the bistatic range) and quickly and unpredictably change their bistatic velocity, unlike the CASA C-295 or PC-12 airplanes.

### 4.5. Cruse Airplane THY9 (DAB Illumination)

**Figure 22:**
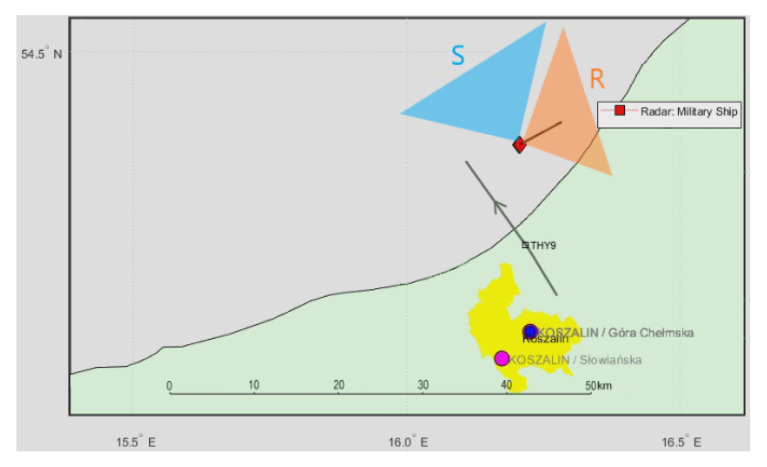
Situation map for the THY9 airplane (Boeing Dreamliner).

**Figure 23:**
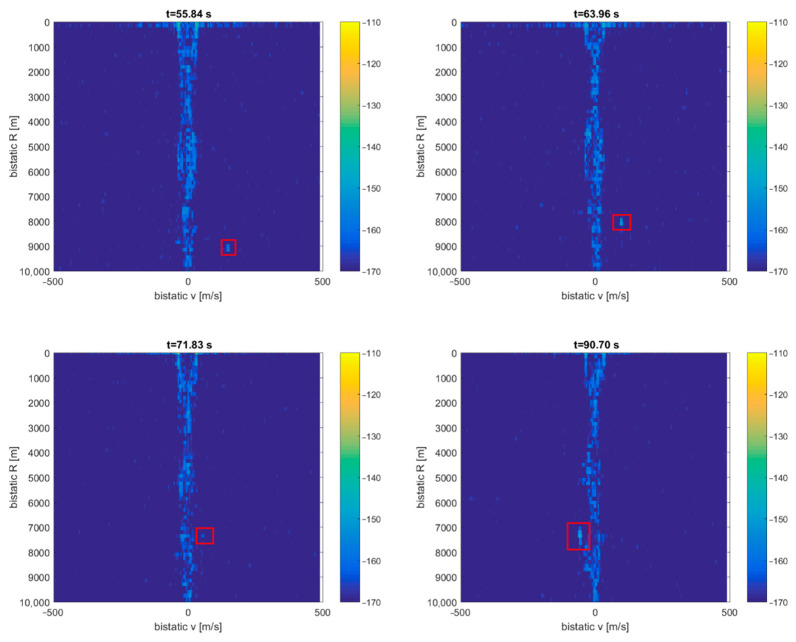
Detection results of the THY9 airplane (subsequent time stamps).

A more in-depth analysis of the acquired signals shows that it is also possible to detect cruise airplanes flying accidentally near the PCL receiver installed on the military ship. Figure 22 shows a situation with the ship sailing northeast slowly and the THY9 [27] cruise airplane (Boeing B789 Dreamliner) flying northwest, ca. 10 km from the ship. The DAB transmitter (218.64 MHz) was selected for illumination, and the vertically polarized antennas with the front reference antenna were employed. Detection results of this cruise flight are shown in Figure 23, with a clear dot traveling in a bistatic *R* = 8 km area and decreasing bistatic velocity. During a period of 77 … 86 s, the echo from the airplane is lost in the clutter area due to low bistatic velocity. This moment correlates very well with the THY9 mark crossing on the transmitter–receiver baseline on the map, as shown in Figure 22.

### 4.6. Cruse Airplane THY79K (DAB Illumination)

**Figure 24:**
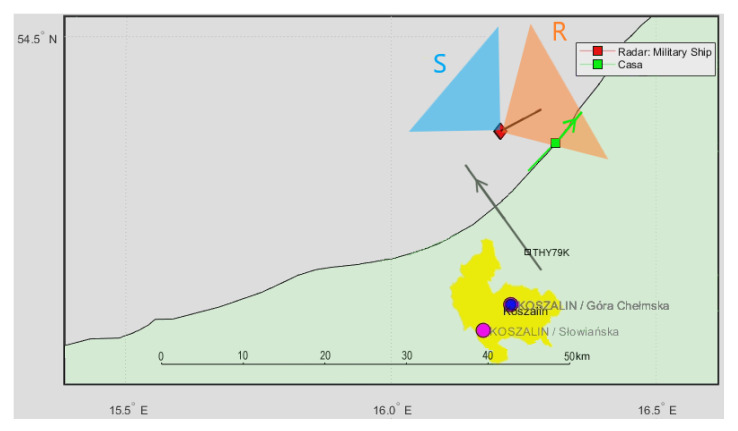
Situation map for THY79K airplane (Boeing Dreamliner) and CASA C-295.

In Figure 24, we can see a very similar situation with another cruise airplane, THY79K (Boeing Dreamliner [28]), flying six minutes later in the same air corridor, and the cooperative CASA airplane (marked with a green square) flying at the coastline, around 5 km from the ship (PCL receiver setup was identical as previously).

**Figure 25:**
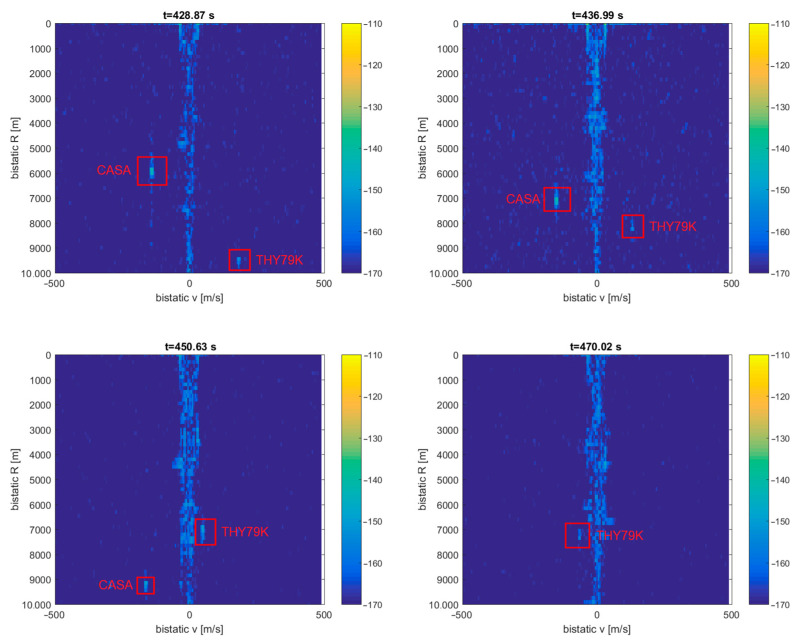
Detection results of the THY79K and CASA airplanes (subsequent time stamps).

In the detection plot (Figure 25), there are clearly visible echoes from the THY79K airplane that was moving on a track located at 7 … 10 km of the bistatic range. The shape and location of this track are very similar to that of THY9 shown in Figure 23. The detection of THY79K is not visible from 454 to 467 s when it falls into the clutter area (bistatic *v* = 0), and this moment correlates with the THY79K mark passing the transmitter–receiver baseline (see Figure 24). Detection of the departing CASA airplane is also visible as a bright point traveling steadily from 6 to 10 km of the bistatic range. It goes beyond the visible area (10 km) after 455 s.

### 4.7. Cruse Airplane LOT4CG (DVB-T Illumination)

**Figure 26:**
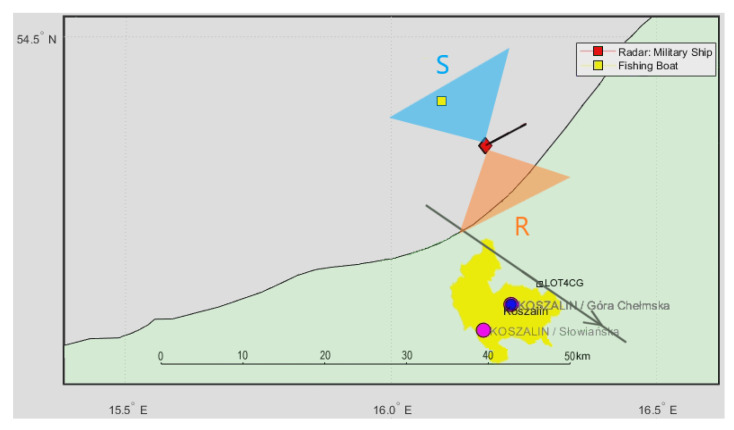
Situation map for the LOT4CG airplane (EMBRAER 175).

In Figure 26, we can see a situation map with the airplane LOT4CG [29] (EMBRAER 175) entering over the land, passing the ship with the PCL receiver, and later the illuminating DVB-T transmitter (191.5 MHz). The horizontally polarized antennas with the side reference antenna were employed for signal acquisition.

**Figure 27:**
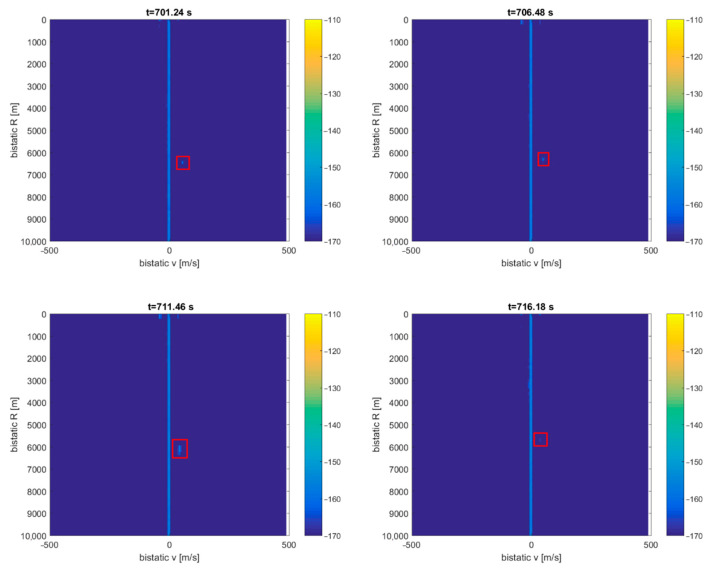
Detection results of the LOT4CG airplane (scene in Figure 26).

**Figure 28:**
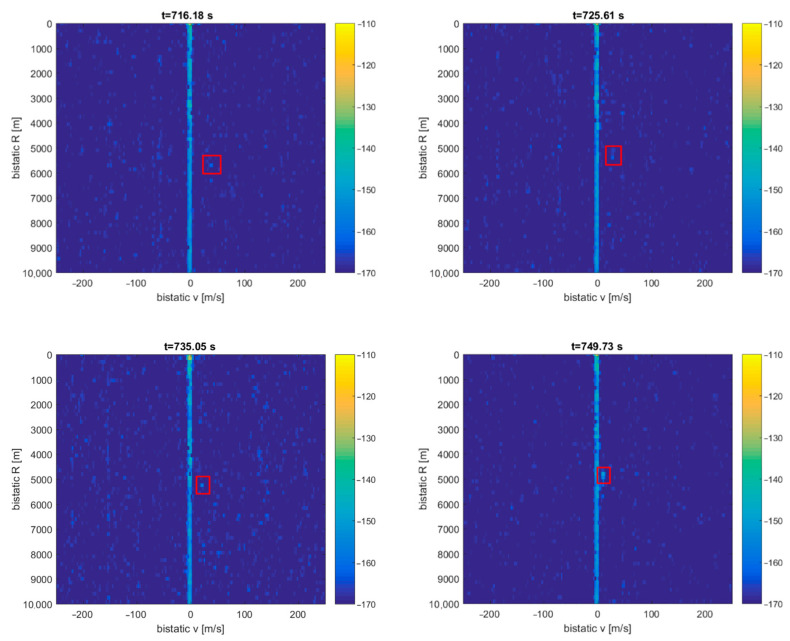
LOT4CG detection visible with integration time increased to 524 ms (scene in Figure 26).

This flight was detected in the bistatic *v*/*R* plane (see Figure 27) as a very weak signal, visible only during a short time (around 29 s). It must be noted that the cross-section area of this airplane is smaller than in the case of Boeing B789, which yields a lower energy of reflected signals. After doubling the integration time to 524 ms, this aircraft’s echo became stronger and visible for about 33 s longer, as shown in Figure 28.

## 5. Conclusions

The presented results clearly show that seaborne passive radar using nearby DAB or DVB-T transmitters can detect airborne targets of different kinds. This allows the system to be used for monitoring the airspace over coastal areas. Such a system can be relatively inexpensive, as it can be built using commercial off-the-shelf VHF antennas, general-purpose SDR receivers, and a computer. Better detection performance can be obtained with more advanced equipment (e.g., more sensitive receivers, higher-gain antennas) and more sophisticated signal processing (e.g., integrating signals from more receiving channels and extending the integration time, which necessitates the use of range/Doppler migration correction techniques).

Maritime targets (such as the fishing boat) were not detected in the conducted experiment. This resulted from the fact that the bistatic velocity of the target was small and the target echo was inseparable from the clutter, which was spread in the velocity dimension. To detect slow targets, such as other ships or boats, longer integration times should be employed, which will increase the velocity resolution. In addition, more advanced clutter removal techniques may be employed, such as STAP (space-time adaptive processing), which provides higher selectivity for unwanted signal attenuation. In general, the problem of clutter removal in seaborne platforms is much more complicated than in the case of stationary ground-based radars. This complication results from the fact that the platform is moving, and an additional Doppler shift is introduced. The clutter characteristics will change depending on the geometry of the transmitter, clutter, and radar, which should be taken into account when the algorithm for clutter cancellation is designed.

## Data Availability

Data sharing is not applicable to this article.
